# Supplementary material for: Deletion of wfs1 Impairs Oligodendrocyte Precursor Cells Dorsal Distribution and Myelination Through the wfs1-hmgcs1 Axis in Zebrafish
Source: Biology (Basel). 2026 Aug 16;15(16):1402. doi: 10.3390/biology15161402 (PMC13510151; doi:10.3390/biology15161402)
Supplement: Supplementary file 1 [file biology-15-01402-s001.zip › File S1-Transcriptome sequencing data and differentially expressed genes (DEGs) in wfs1-- zebrafish at 5 dpf/2.KEGG/wfs1KOvsWT/DOWN/src/dre04150.html]

dre04150


- dre:245702

- Down regulated genes

245702\_1(-1.45433245048201)

- dre:100000252

- Down regulated genes

100000252\_1(-1.7718817168434)

- dre:436718

- Down regulated genes

436718\_1(-6.85571323128554)

- dre:497384

- Down regulated genes

497384\_1(-2.05267237809407)

- dre:324140

- Down regulated genes

324140(-5.90052475401947)

- dre:573492

- Down regulated genes

573492(-2.61487825195395)

- dre:79380

- Down regulated genes

79380\_1(-1.05168581152728)

- dre:797250

- Down regulated genes

797250(-1.06740189529136)

- dre:790938

- Down regulated genes

790938(-4.50740250004449)

- dre:563708

- Down regulated genes

563708(-3.59233336704936)

- dre:359840

- Down regulated genes

359840\_1(-1.77685900658053)

- dre:100333504

- Down regulated genes

100333504\_1(-6.61871930566475)

- dre:336619

- Down regulated genes

336619(-2.2262247407159)

- dre:792689

- Down regulated genes

792689\_1(-1.78857482493481)

- dre:555584

- Down regulated genes

555584(-4.00454883626176)

- dre:792689

- Down regulated genes

792689\_1(-1.78857482493481)

- dre:556087

- Down regulated genes

556087\_1(-6.16688081226611)
- dre:791993

- Down regulated genes

791993(-3.69340367298196)
- dre:30127

- Down regulated genes

30127\_1(-1.48241226857528)

- dre:58072

- Down regulated genes

58072(-6.00118378322126)
- dre:30369

- Down regulated genes

30369\_1(-1.70732701220414)

Close
